# Assessment of virulence diversity of methicillin-resistant *Staphylococcus aureus* strains with a *Drosophila melanogaster* infection model

**DOI:** 10.1186/1471-2180-12-274

**Published:** 2012-11-23

**Authors:** Kaiyu Wu, John Conly, Michael Surette, Christopher Sibley, Sameer Elsayed, Kunyan Zhang

**Affiliations:** 1 Department of Pathology & Laboratory Medicine, University of Calgary, 3330 Hospital Drive NW, Calgary, AB, T2N 4N1, Canada; 2 Department of Microbiology, Immunology and Infectious Diseases, University of Calgary, Calgary, AB, Canada; 3 Department of Medicine, University of Calgary, Calgary, AB, Canada; 4 The Calvin, Phoebe and Joan Snyder Institute for Chronic Diseases, University of Calgary, Calgary, AB, Canada; 5 Centre for Antimicrobial Resistance, Alberta Health Services/Calgary Laboratory Services/University of Calgary, Calgary, AB, Canada; 6 Farncombe Family Digestive Health Research Institute, Departments of Medicine and Biochemistry and Biomedical Sciences, McMaster University, Hamilton, ON, Canada; 7 Department of Medicine, University of Western Ontario, London, ON, Canada; 8 Department of Microbiology & Infectious Disease, University of Western Ontario, London, ON, Canada

**Keywords:** Methicillin-resistant *Staphylococcus aureus*, *Drosophila melanogaster*, Virulence, Infection model

## Abstract

**Background:**

*Staphylococcus aureus* strains with distinct genetic backgrounds have shown different virulence in animal models as well as associations with different clinical outcomes, such as causing infection in the hospital or the community. With *S. aureus* strains carrying diverse genetic backgrounds that have been demonstrated by gene typing and genomic sequences, it is difficult to compare these strains using mammalian models. Invertebrate host models provide a useful alternative approach for studying bacterial pathogenesis in mammals since they have conserved innate immune systems of biological defense. Here, we employed *Drosophila melanogaster* as a host model for studying the virulence of *S. aureus* strains.

**Results:**

Community-associated methicillin-resistant *S. aureus* (CA-MRSA) strains USA300, USA400 and CMRSA2 were more virulent than a hospital-associated (HA)-MRSA strain (CMRSA6) and a colonization strain (M92) in the *D. melanogaster* model. These results correlate with bacterial virulence in the *Caenorhabditis elegans* host model as well as human clinical data. Moreover, MRSA killing activities in the *D. melanogaster* model are associated with bacterial replication within the flies. Different MRSA strains induced similar host responses in *D. melanogaster*, but demonstrated differential expression of common bacterial virulence factors, which may account for the different killing activities in the model. In addition, hemolysin α, an important virulence factor produced by *S. aureus* in human infections is postulated to play a role in the fly killing.

**Conclusions:**

Our results demonstrate that the *D. melanogaster* model is potentially useful for studying *S. aureus* pathogenicity. Different MRSA strains demonstrated diverse virulence in the *D. melanogaster* model, which may be the result of differing expression of bacterial virulence factors *in vivo*.

## Background

*Staphylococcus aureus* is an important human pathogen, causing a wide range of diseases from skin and soft tissue infections to life threatening sepsis [1]. Methicillin-resistant *S. aureus* (MRSA), which causes infections in hospitals and in the community, has become a major public health problem worldwide. MRSA strains can be classified into different clonal groups and subgroups according to their genotypic characteristics. Epidemiologic data have indicated that certain strains are more commonly associated with invasive infections than others [2]. Experimental studies using human neutrophils and a mouse model suggested that community-associated MRSA (CA-MRSA) strains are more virulent than hospital-associated MRSA (HA-MRSA) strains [3]. For CA-MRSA strains, USA300 showed higher virulence than USA400 in a rat pneumonia model [4]. These findings suggest that the virulence of *S. aureus* strains in the animal models may correlate with the clinical outcomes. However, to date, there are 17 major clonal complexes and many more subgroups identified from the *S. aureus* isolates collected worldwide, including MSSA and MRSA strains, and more are expected to be identified [5]. Given this complexity it is difficult to compare the virulence of these strains using mammalian models.

We previously utilized the nematode, *C. elegans,* as a host model to analyze the virulence of major local clinical MRSA isolates, including those belonging to USA300, USA400, and Canadian epidemic strains MRSA 2 (CMRSA2) and CMRSA6. Our results demonstrated that CA-MRSA strains are more virulent than HA-MRSA strains [6]. Moreover, the virulence of MRSA in the *C. elegans* model correlated well with the isolation of MRSA from clinically relevant invasive anatomic sites in humans [6,7], suggesting that the invertebrate model could be a useful tool for studying *S. aureus* pathogenicity.

*Drosophila melanogaster*, the fruit fly, has a number of characteristics which make it a suitable model for studying host interactions with important human pathogens. Drosophila has a complex innate immune system and compared with the innate immunity of *C. elegans*. The fly has the toll and immune deficiency (IMD) signalling pathways that act in response to bacterial and fungal infections, which are homologous to the toll-like receptor (TLR) and tumour necrosis factor receptor (TNFR) pathways in mammals [8]. Drosophila has been used as an infection model for different bacterial species, including *Pseudomonas aeruginosa *[9,10], *Mycobacterium marinum *[11], *Listeria monocytogenes *[12], and *Salmonella *[13]. To date, a few lab strains of *S. aureus* have been analyzed using a fly model and demonstrated virulence [14], suggesting that *D. melanogaster* could be adapted as a convenient high-thoughput model for *S. aureus* infection.

In this study, we employed *D. melanogaster* as a host model to study the virulence of our major local MRSA epidemic strains with different genetic backgrounds. These strains exhibited differing degrees of virulence, with USA300, USA400, and CMRSA2 being more virulent than CMRSA6 and an M92 colonization strain, which correlated with human clinical data and with the *C. elegans* model for these same strains [6]. We observed that the high virulence strains replicated and spread systemically within the fly in a significantly greater manner than they did in the low virulence strains, resulting in greater killing activities. This is thought to be due to greater expression of bacterial virulence factors. Our results suggest that the Drosophila fly model could be another useful invertebrate model for MRSA pathogenesis, and host immunity because of its well characterized innate immune system.

## Methods

### Bacterial strains and growth conditions

The Canadian epidemic MRSA reference strains CMRSA2, 6, 7, and 10 were provided by the National Microbiology Laboratory, Health Canada, Winnipeg, Canada [15]. Strain M92 is a strain which has only been associated with colonization of the nares in hospital staff at our local hospitals, but has not been associated with infection over the course of many years. The clinical isolates used in this study were identified by standard procedures as previously described [6].

### Maintenance of *D. melanogaster* and fly killing assay

*D. melanogaster* Canton S flies were maintained at room temperature on standard cornmeal agar. The feeding assay was performed as previously described [16]. The pricking assay was modified from the method developed by Fehlbaum *et al.*[17]. Briefly, healthy 2–5 day-old female flies were anesthetised on ice and carefully pricked in the dorsal thorax with a 27.5-gauge needle (BD Biosciences) which had been dipped into bacterial cultures adjusted to a concentration of ~8 × 10^8^ CFU/ml in brain-heart infusion (BHI) broth. Fly survival was monitored and recorded from 12 to 72 hours post inoculation. Survival curves were generated by the Kaplan-Meier method, and statistical significance was calculated by log-rank test using Prism 5 (GraphPad Software, Inc.).

### Bacterial *in vitro* growth curve

Overnight bacterial cultures were diluted (1:1000) in fresh BHI broth or M9 minimal salt medium (BD Biosciences), with 200μl loaded onto a 96-well plate. Each well was covered with 50 μl of mineral oil to prevent evaporation. The growth curves of bacterial cultures at 25°C, which mimics the temperature inside fly body, were monitored photometrically by reading the optical density at 600nm using an automatic optical density measuring machine (1420 Multilabel Counter VICTOR, Perkin Elmer).

### Bacterial *in vivo* growth inside flies

Bacterial replication was monitored throughout the fly pricking experiments, and only the live flies were assessed. In order to enumerate viable bacteria in the whole fly at 1, 6, 18, and 24 hours post infection, 8 infected flies were harvested, and the whole flies were homogenized using pestles (DiaMed), and the bacterial number per fly was enumerated. In order to enumerate the bacteria present in specific body parts (*i.e.* crop, head, leg, and wing), 8–10 infected flies were harvested and dissected at 18 hours post infection, with the specific body parts collected into 100μl phosphate buffered saline (PBS) followed by homogenization. The quantitative bacterial counts in the different body parts of each fly were enumerated. For both the whole fly and body part harvesting, the homogenate was re-suspended in 1 ml of PBS, and 100μl of 10-fold serial dilutions were plated onto tryptic soy agar (TSA) with ampicillin (50μg/ml). Colonies were counted following overnight incubation at 37°C. The Mann–Whitney test was performed to determine significant differences between the different strains. For microscopic examination of the whole fly, the infected flies at 18 hours post infection were fixed in 10% neutral-buffered formalin and sent to the Histopathology Laboratory at the Faculty of Veterinary Medicine, University of Calgary, for processing, sectioning, and Gram staining.

### RNA isolation and reverse transcription

For bacterial virulence gene expression *in vitro*, 0.5-ml of bacterial culture at the mid-log phase (OD_600_ ~0.6) and the stationary phase (OD_600_ ~ 4.5 for CMRSA2 and CMRSA6, and OD_600_ ~ 5.0 for USA300, USA400 and M92, based on the bacterial growth curve measurements for each strain) were aliquoted. The total RNA was extracted using TRIzol (Invitrogen). For host antimicrobial peptide (AMP) gene expression or *in vivo* bacterial virulence gene expression, total RNA from five flies chosen randomly at 6, 18, and 24 hours post-infection were extracted using TRIzol, as previously described [18]. Genomic DNA was eliminated by DNase I (Amp Grade, Invitrogen) treatment, and cDNA synthesis was performed with an iScript cDNA Synthesis kit (Bio-Rad, USA).

### Quantitative real-time PCR

qRT-PCR was performed using iQ SYBR Green Supermix (Bio-Rad,USA) on a CFX96 Real-Time Detection System (Bio-Rad, USA). For host gene expression, the thermal cycle conditions were performed as described previously [18]. The expression levels of Drosomycin, Diptericin, and Cecropin A1 at 18 hours post infection in the flies were normalized to the house keeping gene ribosome protein 49 (*rp49*) [18]. For bacterial gene expression, the expression levels of *hla*, *hlg*, *sak*, *ssp*A, and *hys*A in different strains growing in BHI broth at mid-log and stationary phases and inside the flies were normalized to the control gene, *gyr*B, encoding DNA topoisomerase subunit B [19]. All primers used for qRT-PCR are listed in Table 1. Relative target gene expression was calculated according to the ΔΔCt method, in which the fold difference in expression was 2^-ΔΔCt^[20]. The experiments were repeated at least three times. Student’s *t*-test analysis was performed to determine significant differences of the host gene expressions in response to different MRSA strains and the virulence gene expression among different strains.

**Table 1:** Primers used for qRT-PCR analysis

**Primers**	**Sequence (5' to 3')**	**Ref**
*rp*49 F	GACGCTTCAAGGGACAGTATCTG	[18]
*rp*49 R	AAACGCGGTTCTGCATGAG	[18]
*dpt*- F	GCTGCGCAATCGCTTCTACT	[18]
*dpt*- R	TGGTGGAGTGGGCTTCATG	[18]
*dro*-F	CGTGAGAACCTTTTCCAATATGATG	[18]
*dro*-R	TCCCAGGACCACCAGCAT	[18]
*cec*A1-F	TCTTCGTTTTCGTCGCTCTC	[18]
*cec*A1-R	CTTGTTGAGCGATTCCCAGT	[18]
*hla*-F	CTGATTACTATCCAAGAAATTCGATTG	This study
*hla*-R	CTTTCCAGCCTACTTTTTTATCAGT	This study
*hlg*-F	ATAGAAGATATCGGCCAAGG	This study
*hlg*-R	TTGCATCTTAACAACTAGGGC	This study
*sak*-F	GACGCGAGTTATTTTGAACC	This study
*sak*-R	TCTTTTGTAAGTGTAGTCCCAGG	This study
*hys*A-F	GTTTGATGCTACA GAGAAAGAGG	This study
*hys*A-R	CTGCGATTTTCTCAATATTACG	This study
*ssp*A- F	GGGT TATTAGGTTG GTCATCG	This study
*ssp*A-R	AAGTGATCGGAATTCATTGG	This study
*gyr*B-F	ATCGACTTCAGAGAGAGGTTTG	[19]
*gyr*B-R	CCGTTATCCGTTACTTTAATCCA	[19]

## Results

### MRSA strains with greater propensity to cause clinically invasive human infection showed increased fly killing activities

**Figure 1:**
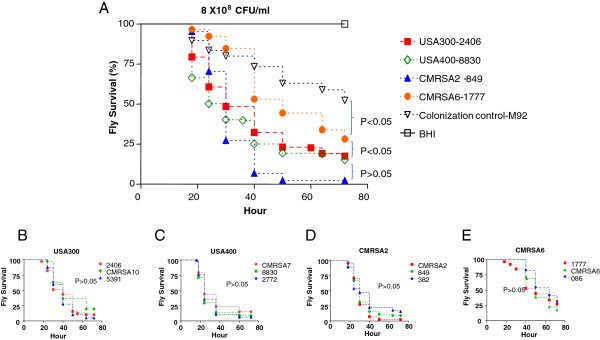
**MRSA strains demonstrated different killing activities against *D. melanogaster. *(A)** Kaplan-Meier survival plots of Drosophila pricked with the representative clinical MRSA strains. (**B-E**) Three clinical isolates within a clonal group demonstrated similar levels of killing activity: **(B)** USA300 isolates (2406, CMRSA10, 5391); **(C)** USA400 isolates (CMRSA7, 8830, 2772); **(D)** CMRSA2 isolates (CMRSA2, 849, 382); **(E)** CMRSA6 isolates (1777, CMRSA6, 086).

We tested both feeding and pricking methods to compare the virulence of clinical MRSA strains in the fly model. Feeding experiments did not show significant differences among these strains in terms of the killing activities (data not shown). However, pricking experiments demonstrated that different clinical MRSA strains had distinct killing activities. Flies injected with plain BHI broth were included as a negative control, for which no flies were killed during the whole period of the experiment. USA300, USA400 and CMRSA2, previously shown to have a greater propensity to cause clinically invasive human infection [6], demonstrated high killing activities, with 51.4%, 60.3% and 72.8% of flies dead at 36 hours, and 83.5%, 84.9% and 97.7% of flies dead at 72 hours, respectively. No significant differences were observed between these strains (p>0.05) (Figure 1A). However, CMRSA6 showed significantly lower killing activity (p<0.05), whereby only 15.3% of flies died at 36 hours and 71.8% at 72 hours. Moreover, the colonization strain M92 showed significantly lower killing activity compared with CMRSA6 (p<0.05). To further confirm the differential fly killing activities described above, two additional clinical isolates from each clonal group with similar genetic backgrounds were tested. It was noted that all isolates belonging to the same clonal group demonstrated similar killing activities (p>0.05) (Figure 1B-E). However, all the members of each clonal group from USA300, USA400 and CMRSA2 showed significant differences to all the members of CMRSA6 group (all p<0.05), but no significant differences were observed between all the strains of each clonal groups from USA300, USA400 and CMRSA2 (all p>0.05). Taken together, these results confirmed that USA300, USA400, and CMRSA2 strains were highly virulent in the fly model, while CMRSA6 and M92 were considered to be of lower virulence.

### MRSA proliferation and dissemination correlated with fly killing activity

We have observed that USA300, USA400, and CMRSA2 were more virulent than CMRSA6 and M92 in the fly model. To investigate whether the growth rate inside the flies was associated with the fly killing activity, we measured the bacterial growth *in vitro* (M9 minimal medium and BHI broth, 25°C) and *in vivo* (inside the fly).

**Figure 2:**
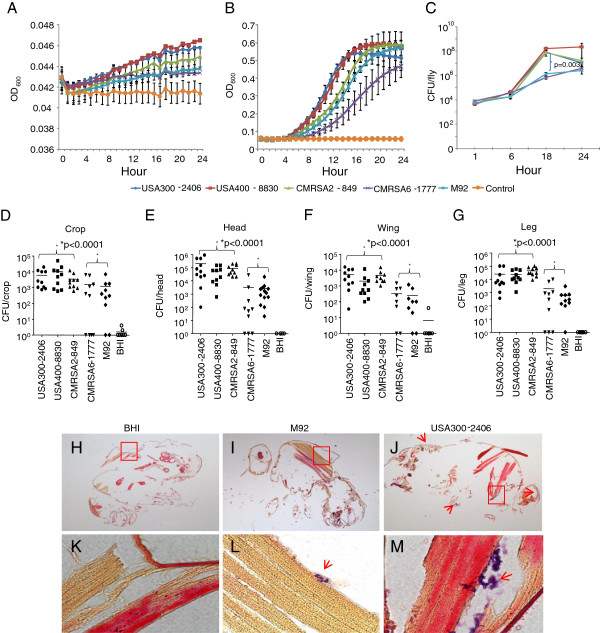
**MRSA proliferation correlated with fly killing activity.** Growth curves of MRSA strains in M9 minimal medium **(A)** and brain heart infusion (BHI) broth **(B)** at 25°C for 24 hrs. **(C)** Growth of MRSA strains within the flies for 24 hrs. A batch of live flies was harvested at 1, 6, 18, and 24 hours post infection and CFU/fly was determined. **(D-G)** Bacterial counts in different body parts from the flies infected with different MRSA strains at 18 hours post infection: **(D)** crop; **(E)** head; **(F)** wing; **(G)** leg. The asterisk indicates a statistically significantly difference (p < 0.05) between groups of the high virulence strains and the low virulence strains in bacterial counts in different body parts (Mann–Whitney test). **(H-M)** Microscopic examination of representative histopathological sections of BHI broth-injected (control) flies **(H,K)**, and M92 **(I, L)** and USA300-2406 **(J, M)** infected flies, low (4X) and high magnification (100X) respectively.

The high virulence strains USA300 and USA400 had the highest growth rates in both BHI broth and M9 minimal medium; but CMRSA2 had a lower growth rate and similar virulence to USA300 and USA400 in the fly model (Figure 2A and B), indicating that the growth rate *in vitro* was not associated with virulence in the fly model. On the other hand, *in vivo* results indicated that the high virulence strains had a higher growth rate than the low virulence strains *in vivo*. At 1 hour post infection, similar bacterial counts (0.43 × 10^4^ to 0.83 × 10^4^ CFU/fly) were observed for all MRSA strains (Figure 2C). The bacterial counts per fly increased by time indicating that bacterial replication was occurring and 1.8 × 10^4^ - 4.2 × 10^4^ CFU/fly were observed for all strains at 6 hours. Following the 6 hour mark, the high virulence strains, USA300, USA400 and CMRSA2, grew exponentially and the viable bacterial counts were 0.77 × 10^8^-1.7 × 10^8^ CFU/fly by 18 hours. The low virulence strains grew more slowly and by 18 hours the viable bacterial counts were 0.72 × 10^6^ CFU/fly for CMRSA6 and 1.4 × 10^6^ CFU/fly for M92. A significant difference was observed between the high virulence strains and the low virulence strains (p=0.003). At 24 hours post infection with the high virulence strains, dead flies were excluded from the experiment. With the surviving flies, the viable bacterial concentration per fly was approximately 10^7^ CFU/fly for USA300 and CMRSA2 infected flies, and 10^8^ CFU/fly for USA400. With CMRSA6 and M92 infected flies, the bacterial counts were about 3.0 × 10^6^ CFU/fly at 24 hours.

We further investigated whether the growth rate inside flies was associated with bacterial dissemination within the fly, or with a localized infection, depending on the strain of MRSA. The bacterial loads in different body parts (*i.e.* crop, head, wing and leg) of flies infected with the high and low virulence strains were determined. We found that bacterial cells were present in all body parts for all strains. However, the low virulence strains had lower numbers of bacteria in each body part compared to the high virulence strains. In the crops, more bacteria were observed in USA300 (6 × 10^3^ CFU/crop), USA400 (1.1 × 10^4^ CFU/crop), and CMRSA2 (3.5 × 10^3^ CFU/crop) infected flies than CMRSA6 (1.6 × 10^3^ CFU/crop) and M92 (1.2 × 10^3^ CFU/crop) infected flies at 18 hours post infection. Similarly, there were higher numbers of USA300, USA400 and CMRSA2 (>3.3 folds) compared with CMRSA6 and M92 in the head, leg, and wing (Figure 2D-G). There were significant differences (p<0.0001) between the groups of the high virulence strains and the low virulence strains in terms of the bacterial load in these body parts.

To further demonstrate the difference in the *in vivo* growth rates between the high virulence and low virulence strains, we examined the flies infected with USA300-2406 (high virulence) and M92 (low virulence) by histopathology. As shown in Figure 2H-M, the M92 infected fly had a small number of Gram positive cocci in the dorsal thorax, while the USA300 infected fly had significantly greater concentration, as well as in other body parts compared with the M92 infected fly and the controls.

### Host innate immune response to MRSA infection

**Figure 3:**
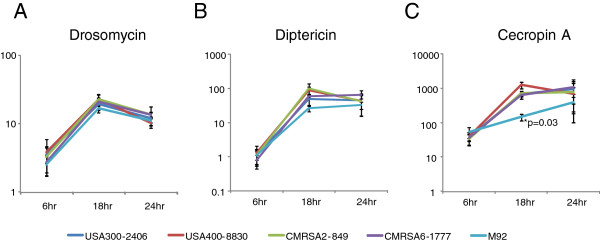
**Host immune responses to MRSA infection.***D. melanogaster* AMP gene induction at 6, 18 and 24 hour post infection was calculated by qRT-PCR as fold change of the transcriptional level in the MRSA infected flies relative to the BHI broth-injected flies: **(A)** Drosomycin induction; **(B)** Diptericin induction; **(C)** Cecropin A1 induction. The asterisk indicates a statistically significantly difference (p = 0.03) between M92 and other MRSA strains in inducing host Cecropin A1 expression at 18 hours post infection (Student’s *t*-test).

Drosophila mounts innate responses following bacterial challenge by secreting different antimicrobial peptides (AMPs), such as drosomycin, diptericin, and cecropin A1. We measured the fly host immune response to different MRSA strains in order to determine whether this response correlates with the observed fly killing activity. The induction of drosomycin, diptericin and cecropin A1 in the infected flies was shown as a fold change of transcriptional level relative to the constitutive transcriptional level of these genes in control flies pricked with BHI broth. For all strains, the transcription of all three AMPs was activated post infection. No significant difference in drosomycin or diptericin gene expression was observed among the flies infected with the various strains. (Figure 3A and B). There was a marked difference noted for cecropin A1 gene expression among the various strains. The transcriptional level increased 37- to 54-fold for all flies 6 hours post infection, and 146 to 1253-fold at 18 hours (Figure 3C). At 18 hours, the transcriptional level of cecropin A1 was 146-fold higher in the M92-infected flies than the control flies, which was significantly lower than the fold increase seen in the flies infected with the other strains (642–1253 fold, p=0.03). This difference was also observed at 24 hours post infection, although no statistical difference was observed. Our results demonstrated that different MRSA strains induced similar levels of fly innate immune responses except for M92 which induced much less cecropin A1.

### Different MRSA strains have distinct bacterial virulence gene expression patterns

Since different MRSA strains induced similar host responses, we determined whether the differences in *S. aureus* virulence seen in the fly model could be accounted for by differing bacterial virulence gene transcriptional levels. We compared the transcriptional levels of 5 common virulence genes using qRT-PCR. These genes included 2 haemolysins (hemolysin α and γ; *hla* and *hlg*) and 3 exoenzymes (hyaluronidase, staphylokinase, and V8 protease; *hys*A, *sak* and *ssp*A) in MRSA strains using qRT-PCR. Due to the fact that the quantity of RNA was low at 6 hours and most flies were dead at 24 hours post infection, only bacterial RNA at 18 hours was harvested.

**Figure 4:**
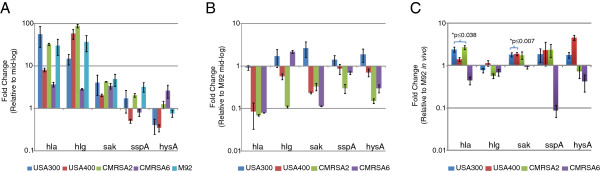
**Comparison of 5 virulence gene expression profiles between different MRSA strains. (A)** Fold-change in the transcriptional level for each gene in MRSA at stationary phase relative to the level in bacteria at mid-log phase *in vitro* (BHI broth); **(B)** Fold-change in the transcriptional level for each gene of MRSA strains relative to the level of M92 at mid-log phase *in vitro* (BHI broth); **(C)** Fold-change in the transcriptional level of each gene in MRSA strains relative to the level of M92 at 18 hour in the flies post infection (*in vivo*). The asterisk indicates a statistically significantly difference (p < 0.05) of the *in vivo* virulence gene expression in the MRSA strains as compared with M92 (Student’s *t*-test). Hemolysin α (*hla*): USA300 vs M92, p=0.0013; USA400 vs M92, p=0.038; and CMRSA2 vs M92, p=0.0015. Staphylokinase (*sak*): USA300 vs M92, p=0.006; USA400 vs M92, p=0.007; CMRSA2 vs M92, p=0.0698.

The first comparison that was made for virulence gene expression was between the mid-log and stationary phases of bacteria grown in BHI broth. The expressions of *hla*, *hlg* and *sak* were higher in the stationary phase than in the mid-log phase for all strains (Figure 4A), which is consistent with previous studies [21-23]. The expressions of *ssp*A and *hys*A were higher in the mid-log phase for some strains, suggesting that the expression of these genes varied among strains. We subsequently compared the virulence gene expression of *S. aureus* strains against that of M92 *in vitro* (Figure 4B). All strains were found to have lower *hla* expression than M92 *in vitro*, but varied in the expression of other genes, with no specific pattern noted. When *in vivo* virulence gene expression was examined, it was noted that *hla* expression was significantly higher in all high virulence strains (USA300, USA400 and CMRSA2; p values: 0.0013, 0.038 and 0.0015, respectively) but not in the low virulence strain CMRSA6 as compared with M92 (Figure 4C). High *in vivo* expression of *sak* and *sspA* were also observed in the high virulence strains but not all of them exhibited significant difference (*sak*, p values: 0.006, 0.007 and 0.0698 for USA300, USA400 and CMRSA2, respectively; *sspA*, all p > 0.05) (Figure 4C). The other genes displayed different gene expression patterns in different strains without correlation with fly killing activity. CMRSA6, a low virulence strain, showed lower *in vivo* gene expression compared with M92 for all genes tested.

## Discussion

Needham and co-workers [14] have shown that a limited number of *S. aureus* lab strains caused fly death following injection of bacteria into the dorsal thorax of the flies, suggesting it is a useful model for high-throughput analysis of *S. aureus* virulence determinant. In this study, we compared the virulence of MRSA strains with different genetic backgrounds using the fly model and demonstrated that they had different fly killing activities, where USA300, USA400, and CMRSA2 strains had greater killing activities compared to CMRSA6 and M92. We had previously described the nematocidal activity of these strains in the *C. elegans* host model, in which USA300, USA400, and CMRSA2 were demonstrated to be virulent, but CRMSA6 and M92 were non-virulent. [6]. The results from this study further support the notion that innate immunity is conserved between *C. elegans* and *D. melanogaster*. *C. elegans* and *D. melanogaster* are evolutionarily closely related and have been shown to possess homologous proteins in the innate immunity, such as p38 MAPK [24],

It has been demonstrated that *P. aeruginosa* is capable of invading and degrading fly tissues, possibly utilizing the fly tissues as a nutrient source [25]. For *S. aureus*, it induces systemic infection in the flies following injection into the dorsal thorax, wherein *S. aureus* cells were found to be present throughout the body of the fly, followed by fly death [14]. In this study we demonstrated that the low virulence strains were limited to a localized infection, but the high virulence MRSA strains proliferated and spread systemically compared with the low virulence strains. We noted that the growth rate *in vivo* does not correlate with that *in vitro*, either in rich or minimal medium (Figure 2A-C). Bacterial counts in various fly body parts, as well as Gram staining and microscopic examination revealed that less than 1% of the entire bacterial load was seen in these different body parts suggesting that most bacteria were probably still located near or outside the injection sites of the dorsal thorax, and bacteria likely entered the circulatory system and subsequently spread to the different fly organs. However, compared with the low virulence strains, significantly more bacterial cells were observed in the organs and tissues of the flies infected with the high virulence strains. This observation is further supported by microscopic and histopathological examination of the whole fly. It is possible that the bacteria encountered the host AMPs and phagocytes, and that the immune response was capable of inhibiting proliferation and further spreading of the low virulence strains compared with the high virulence strains. It was also noticed that two low virulence strains, CMRSA6-1777 and M92 have the same *in vivo* growth but different virulence, which needs to be further investigated in the future studies. For CMRSA2-849, which had the highest cfu counts and caused the most deaths after 72 hrs, the killing mechanisms may be more complex.

To better understand the host-pathogen interactions, we assessed the host immune response to MRSA strains having different genetic backgrounds. *D. melanogaster* has a well described innate immune system and activation of the toll and the immune deficiency (IMD) signalling pathways by infection leads to synthesis of AMPs. These small peptides are primarily produced in the fat body and secreted into the hemolymph [26]. AMPs have various properties, including microbicidal activity against Gram-negative bacteria, Gram-positive bacteria, and/or fungi. It has been shown that diptericin and cecropin are active against Gram-negative bacteria while drosomycin is active against fungi. However, one study showed that in the wild type flies, *S. aureus* elicited a strong induction of AMP genes, including cecropin A, drosomycin, and diptericin [27]. This study demonstrated that MRSA strains with different genetic backgrounds are capable of inducing the expression of these genes, with the highest expression level at 18 hours, and with a decrease or stabilization at 24 hours. The high virulence strains did not suppress AMP gene expression, but rather induced AMP gene expression to the same extent that low virulence strains did. This finding is in contrast to previous observations in a *P. aeruginosa* – *D. melanogaster* infection model whereby a virulent *P. aeruginosa* strain suppressed or poorly elicited AMP gene expression, while the avirulent strain induced gene expression [28]. In the current study, the low virulence strain, M92, induced significantly less cecropin A1 expression at 18 hours post infection compared with the other strains (Figure 3C) even though M92 and CMRSA6 are both the low virulence strains. As described earlier, M92 is a colonization strain, isolated from health care workers and has never been associated with infection. This strain may have developed the ability to tune down the host immune response thereby facilitating colonization rather than clearance by the host. Alternatively, this strain may have lost virulence factors associated with inducing high levels of cecropin A1 in the flies. The mechanism for this observation requires further study.

The mechanisms contributing to the virulence of *S. aureus* are likely determined by the genetic background of each strain as well by the specific combination of virulence genes. Previously, we have determined the presence of 34 virulence genes studied by PCR in MRSA strains, but no specific genes that were directly associated with the hypervirulence of USA300, USA400, and CMRSA2 were identified [6]. The different virulence between these MRSA strains in the fly model may have resulted from differential bacterial virulence gene expression, as Loughman *et al.* have shown that differential bacterial virulence gene expression can be associated with different clinical outcomes during human infections [29]. In this study we determined the *in vitro* and *in vivo* expression levels of 5 common bacterial virulence genes, including 2 hemolysins (*hla* and *hlg*) and 3 exoenzymes (*sak*, *hys*A and *ssp*A), involved in invasive *S. aureus* infection. Our results agreed with previous studies that *hla*, *hlg*, and *sak*, had higher gene expression levels in the stationary growth phase for all strains (Figure 4A) [21-23]. Other studies also noted that *ssp*A was expressed more in the stationary phase [30], while *hys*A was expressed to a lesser degree [31]. Our results showed that the expression levels of *ssp*A and *hys*A differed in the individual strains (Figure 4A), suggesting that regulation of these gene varies between strains, which could be related to the specific genetic background. Further comparisons demonstrated that the expression of *hla in vivo* was significantly higher in all high virulence strains compared to both low virulence strains although the opposite results were observed *in vitro* (Figure 4B,C). Hemolysin α has been implicated as one of the most important virulence factors for *S. aureus*[32], not only in forming pores on the host cell membrane, but also in inducing the release of cytokines and chemokines [33]. Vaccination against hemolysin α showed efficient protection for mice in a *S. aureus*-induced pneumonia model [34,35]. A recent study also demonstrated that hemolysin α contributed to severe skin infection caused by a USA300 strain in a mouse model, and that vaccination against hemolysin α provided efficient protection in this model [36]. Collectively, previous studies and our results suggest that killing activity in the fly model arises from the interplay of multiple virulence factors, with hemolysin α being one of the major factors contributing to the virulence in the model. However, this hypothesis requires confirmation in future studies. Additionally, it is necessary to point out that the fly model is still an invertebrate model and the virulence in the fly model may not necessarily reflect the virulence in human infection. For example, as shown in a previous study [14], *agr* and *sar* mutants, which have reduced virulence in mammalian models [37,38], did not show significantly attenuated virulence in the fly model.

## Conclusions

Our results demonstrated that the *D. melanogaster* model was a useful model for studying the virulence of MRSA, as MRSA strains with the distinct genetic backgrounds had different degrees of virulence in the *D. melanogaster* model, which may have resulted from the differential expression of bacterial virulence factors *in vivo*. These results are similar to what we observed in the *C. elegans* model and, therefore, the fly represents another model for the high-throughput analysis of *S. aureus* virulence. We believe the information obtained from this study provides new insights into the interactions between bacteria and the host, but we recognize more studies will be needed to elucidate the killing mechanism in the fly model.

## Authors' contributions

KW and KZ conceived the idea and designed the overall study. KW performed experiments. JC, MS, CS and SE contributed to the experimental design and the analyses of the experimental results. JC and KZ supervised the overall study. KW and KZ prepared the manuscript. All authors have read, commented and approved the final manuscript.
